# Trellis-forming stems of a tropical liana *Condylocarpon guianense* (Apocynaceae): A plant-made safety net constructed by simple “start-stop” development

**DOI:** 10.3389/fpls.2022.1016195

**Published:** 2022-12-19

**Authors:** Patricia Soffiatti, Emilien Fort, Christine Heinz, Nick P. Rowe

**Affiliations:** ^1^ Department of Botany, Federal University of Parana State, Curitiba, Brazil; ^2^ AMAP, Univ. Montpellier, CNRS, CIRAD, INRA, IRD, Montpellier, France

**Keywords:** lianas, trellis, biomechanics, anatomy, development, lianoid wood, safety, mechanical perturbation

## Abstract

Tropical vines and lianas have evolved mechanisms to avoid mechanical damage during their climbing life histories. We explore the mechanical properties and stem development of a tropical climber that develops trellises in tropical rain forest canopies. We measured the young stems of *Condylocarpon guianensis* (Apocynaceae) that construct complex trellises *via* self-supporting shoots, attached stems, and unattached pendulous stems. The results suggest that, in this species, there is a size (stem diameter) and developmental threshold at which plant shoots will make the developmental transition from stiff young shoots to later flexible stem properties. Shoots that do not find a support remain stiff, becoming pendulous and retaining numerous leaves. The formation of a second TYPE II (lianoid) wood is triggered by attachment, guaranteeing increased flexibility of light-structured shoots that transition from self-supporting searchers to inter-connected net-like trellis components. The results suggest that this species shows a “hard-wired” development that limits self-supporting growth among the slender stems that make up a liana trellis. The strategy is linked to a stem-twining climbing mode and promotes a rapid transition to flexible trellis elements in cluttered densely branched tropical forest habitats. These are situations that are prone to mechanical perturbation *via* wind action, tree falls, and branch movements. The findings suggest that some twining lianas are mechanically fine-tuned to produce trellises in specific habitats. Trellis building is carried out by young shoots that can perform very different functions *via* subtle development changes to ensure a safe space occupation of the liana canopy.

## Introduction

Vines and lianas are well known for their climbing life histories that differ radically from self-supporting trees. Young shoots often develop stiff properties and exhibit circumnutational movements that search for supports (Darwin, 1867; Speck and Rowe, 1999). Mature and old stems of lianas are well known for their compliant, flexible (Speck and Rowe, 1999), and fracture-resistant properties (Fisher and Ewers, 1989; Putz and Holbrook, 1991). A key adaptive trait of vines and lianas compared with shrubs and trees is the economy of design where the climbing stem remains slender but can still reach the well-lit surface of the forest canopy *via* the mechanical support afforded by trees. A potential downside of this strategy is that slender stems are at risk of mechanical failure or detachment by the swaying movements caused by wind action or branch and tree falls. In fact, as soon as a climbing plant attaches irreversibly to the stem or branches of a tree, it needs to be able to withstand the mechanical forces that are prevalent in natural habitats, causing the tree and its branches to sway. Numerous reports in the literature have characterized the mechanical and organizational diversity of the young searcher phase and the older compliant more flexible stage (Speck and Rowe, 1999). However, not much is really known about the transition point from stiff to compliant properties and what exactly produces this transition.

In many climbing plants, the attachment organ itself can protect the climbing plant from damage or catastrophic falls from trees. For example, in rattan palms, the long cirri (modified spiny leaf rachises) or flagella (modified spiny fertile axes) can ratchet and attach onto a more apical series of spines if the connection under tension becomes too tight or a swaying movement momentarily separates the climber from the branches of the host tree (Putz, 1990; Isnard and Rowe, 2008). In the Boston ivy, Parthenocissus tricuspidata, adhesive pads borne on branched tendrillar organs can fail in a sequential way when loaded and thus potentially avoid catastrophic failure of the climbing stem from the support, as the whole attachment structure will not fail as a whole (Steinbrecher et al., 2010). In climbing plants that attach by stem twining such as *C. guianense*, the transition from stiff to compliant stem properties is known to occur (Rowe and Speck, 1996), but, up to now, we do not know precisely when.

**Figure 1:**
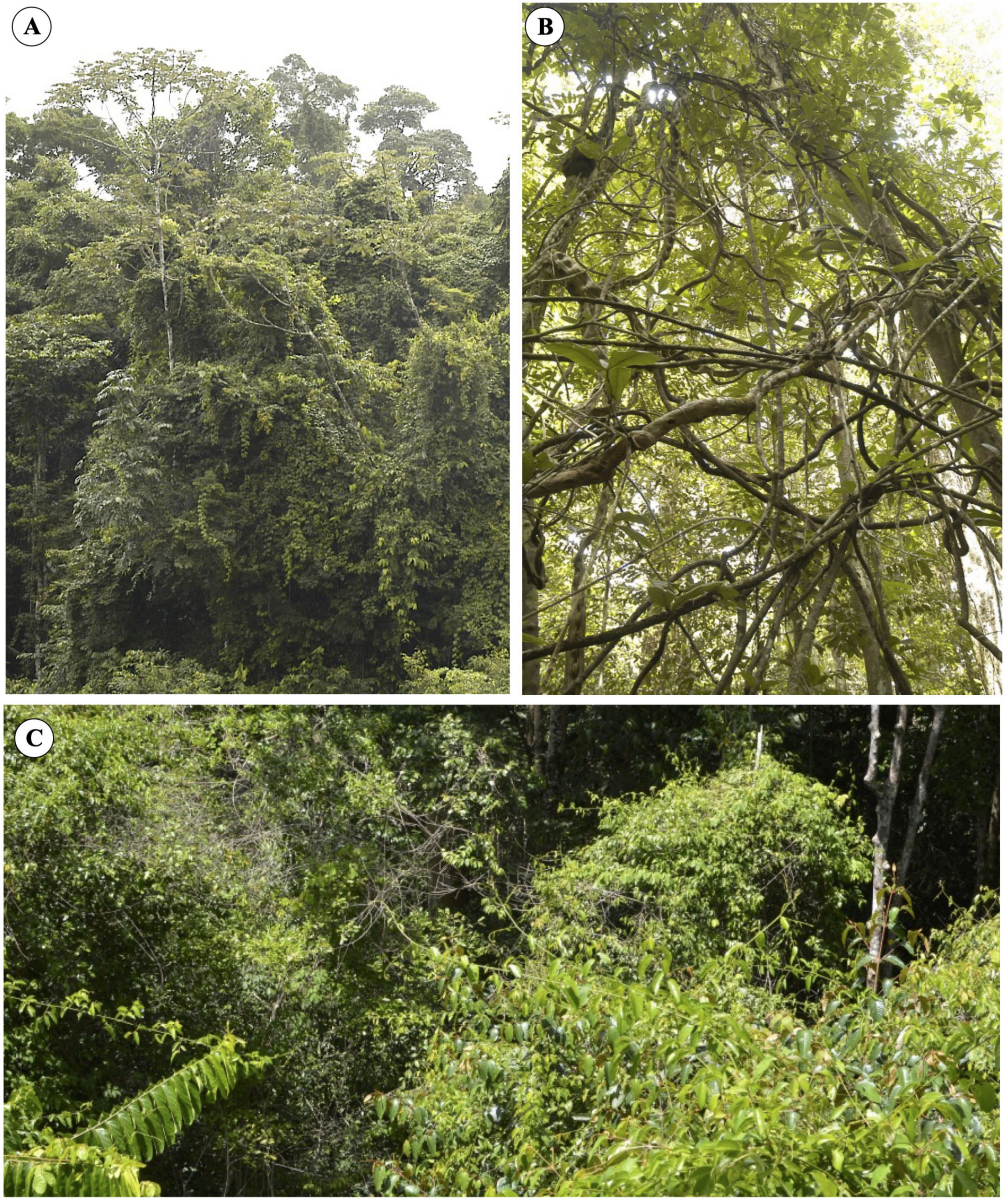
Liana trellis formations in lowland tropical rainforest of French Guiana. **(A)** Overview of a forest margin where the young liana stems form an extensive trellis, which blankets the branches of most trees. A number of young shoots possess leaves and trail or hang down. **(B)** An old trellis formation in the forest understory. The interconnected stems have now matured and are probably composed of highly compliant and tough stem properties. The trellis has probably undergone significant slips and partial falls because of tree falls and wind action but is still living and supporting leafy stems in the canopy above. **(C)** A view of liana trellises on the surface of the tropical rain forest canopy. Entire tree canopies are completely covered by leafy inter-attached stems. Other young shoots “searchers” are crossing gaps and linking up to neighboring trees.

The so-called liana trellises are especially common in tropical humid forests. In the absence of a suitable host or to cross gaps, twining species can self-form networks and trellises that possibly help them cross gaps (Gallentine et al., 2020), but this remains to be fully understood. They form *via* interconnected networks of young to old stems of the same species (self-forming trellis) or interconnected networks of climbing plant stems and host plant stems (**Figures 1A–C**). Many vines and lianas possess invasive traits where young shoots can rapidly attach and smother or carpet host vegetation (Putz, 1990; Schnitzer et al., 2015).

Liana trellises represent an excellent opportunity to study how young shoots of lianas make the transition from stiff unattached self-supporting searchers to attached compliant and flexible stems. Trellises represent areas to sample where young to older shoots are abundant and where the growth and light conditions are similar. Previous studies have determined the overall changes in anatomy and stem mechanics during its life history (Rowe and Speck, 1996); made comparisons of this species with other phylogenetically and morphologically diverse climbers (Rowe et al., 2004); analyzed the chemical composition and ultrastructural organization of the early stiff and later compliant types of wood (Hoffman et al., 2003) and most recently compared the length and reach of searcher stems with other diverse climbing plants (Hattermann et al., 2022). An early study (Rowe and Speck, 1996) demonstrated that anatomical and mechanical properties of young trellis shoots could vary. The study mentioned how mechanical properties of shoots and young branches of the trellis potentially varied according to the exact habit and growth behavior of different young shoots including self-supporting, attached, and hosting support within a trellis of the same individual (Rowe and Speck, 1996).

*Condylocarpon guianense* (Apocynaceae) is a common woody climbing plant (liana) in lowland humid forest of French Guiana (Mori et al., 1997). It climbs by twining of the main stem on host supports and is common as trellis structures both in the tree canopy and forest margin. It readily forms trellises on host vegetation (trees) and produces conspicuous searcher shoots on forest edges and disturbed marginal areas. Mature stems of *C. guianense* are common in understory habitats as flexible compliant stems that often reach diameters greater than 20 cm. In this paper, we analyze the mechanical properties and tissue development of trellis shoots of *C. guianense*. We aimed to explore the development of trellis shoots during the key transition phase from the self-supporting searcher phase to fully attached stems. This is a crucial adaptive transition in terms of stem mechanics for ensuring adequate safety of young trellis stems vulnerable to mechanical failure because of swaying and movements of host branches. We aimed to address the following questions:

1) What is the likely trigger of the switch from stiff to flexible wood? Is it adaptive and does it occur because of attachment?
2) To what extent does the transition influence stem stiffness and what are the modifications in tissue organization that underlie these changes in mechanics?
3) Is there a threshold for the maximum diameter and potential length and reach of young stiff searcher shoots? In addition, what happens if trellis shoots do not find a support?
4) Is the transition from stiff to compliant reversible? Can trellis shoots become stiff again after attachment and the onset of compliant wood?

Finally, because of their ability to navigate unpredictable and unstructured environments safely, climbing plants are of increasing of interest for new bioinspired technologies in materials science and robotics. We discuss the potential of the twining habit and trellis-forming behavior for some of these new bioinspired applications.

## Materials and methods

### Material and location

We targeted a forest margin environment for studying the liana trellis on the Piste de St. Elie approximately 1 km SW of the IRD (Institut de Recherche pour le Développement) research camp facility (5°30′N, 53°00′W) about 140 km northwest of the main town Cayenne.

### Sampling of material

**Figure 2:**
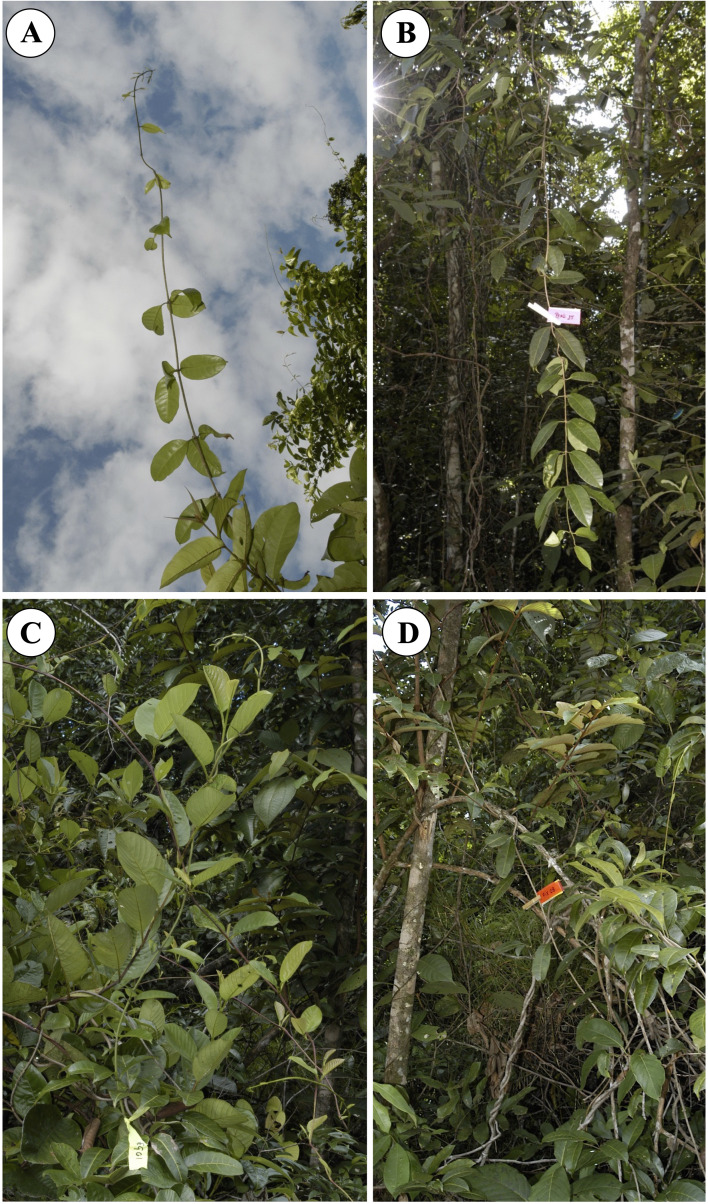
Four phases of stem growth that make up a trellis and which were sampled for mechanical properties and structural development. **(A)** Self-supporting stem, a young stem that has not yet encountered a support. **(B)** Pendulous stem that has not encountered a support. **(C)** Climbing stem represented by a young shoot, which has encountered a support and which has developed twining on that support. **(D)** A “fixed stem”, a stem that shows the upper and lower phases of twining so that the segment in between the two attachment points is completely fixed between two sections of twining onto a host stem.

Because of their often clonal life-history and haphazard stem proliferation in forest margin environments, it is particularly problematic to sample liana stems and to be sure of sampling truly independent individuals rather than clonally derived ramets. We sampled four different shoot categories: (i) self-supporting shoots (25 individuals; 52 stem segments) that had not yet attached to any support (**Figure 2A**); (ii) pendulous shoots (25 individuals; 89 stem segments): stems that had not found an attachment point and which were no longer self-supporting, such stems were often leafy (**Figure 2B**); (iii) attached climbing stems (25 individuals; 63 stem segments) trellis stems that had attached to a support near or below the growing apex of the shoot (**Figure 2C**); (iv) fixed stems (25 individuals; 65 stem segments): trellis stems that were securely twined around a support near the base of a segment and twined around a support near the apex of the segment (**Figure 2D**). All categories of stem were pruned from the trellises composed of host trees and stems of *C. guianense* of the forest margin that extends for some 200 m along the forest margin. Sampled stems were selected at least 5 m apart, thus reducing but not eliminating the possibility that sampled shoots were derived from the same individual or same branch system of an individual. A similar sampling strategy was applied for sampling diverse twining lianas (Lehnebach et al., 2022) and insured that the sampling regime remained in a similar environment and did not vary radically in terms of light and other factors.

Self-supporting stems were sampled including stems that naturally maintained a self-supporting habit. To ascertain this in the field, upright to horizontal stems were cut at their base and then held at this point to see whether the stems showed deflection to a resting position or whether they held their orientation as mechanically stable cantilevers. Stem segments up to 1.5 m in length were pruned from the trellis with sharp secateurs, the cut end placed in water, and the shoots carefully sealed within plastic bags. Shoots were transported to the field laboratory, kept in cool, shaded conditions, and underwent mechanical tests within 48 h.

### Mechanical tests

Stem segments were prepared for three-point bending tests and followed the field laboratory protocol reported by Ménard et al. (2009). Some of the shoots, particularly climbing and fixed shoots, were not always straight and were problematic to test in the preferred four-point bending. Instead, the three-point bending tests were carried out on all specimens. To ensure that the results included pure bending rather than including significant deflection by shear, the so-called span tests were carried out on basal, medium, and apical portions of each category of shoot to find the optimal span-to-depth ratio for the young to old segments of the species shoots (Vincent, 1990; Rowe et al., 2006). We obtained a span-to-depth ratio of 40 after span-to-depth tests, and all specimens were tested in three-point bending as close as possible to this. Bending measurements were carried out on a manual bending apparatus, where a stem segment is placed bridging two supports with the pre-determined suitable span-to-depth ratio (Rowe et al., 2006). Five to six weights were placed at 30-s intervals manually onto a pannier suspended from the exact middle of the specimen to give a suitable deflection of the specimen within the elastic limit, usually 1 to 3 mm depending on the thickness and length of the specimen. The deflection of the specimen was visualized *via* a binocular microscope mounted on the apparatus with an eyepiece graticule. Flexural stiffness (*EI*, Nmm^2^) was calculated from the force/displacement curve generated by adding the sequence of weights


(Eq. 1) $$\text{Stem rigidity},EI_{axis}=l^{3}b/48$$

where *l* represents the span distance (mm) between the two supports and *b* represents the slope of the force (N)/deflection (mm) curve.

The second moment of area of the stem approximated as an ellipse was measured *via* the following formula:


(Eq. 2) $$I_{axis(ellipse)}=(\pi /4)a^{3}b$$

where *a* and *b* represent the mean radial widths of the vertical (*a*), in the direction of the applied force, and horizontal (*b*) directions (measured at the center of the stem and 25% distally and proximally from the center).

The stiffness, Young’s modulus (MPa), in bending was measured *via* the following formula:


(Eq. 3) $$E_{entireaxis}=EI_{axis}/I_{axis(ellipse)}$$

One to seven (typically, one to three) bending tests were carried out on randomly selected branches of each stem category. A variable number of bending tests per specimen was unavoidable between randomly selected individuals, because many stems varied in length, diameter, and included segments that could not be tested properly because of curved or twisted segments.

### Anatomical analysis

Following mechanical tests at the nearby Paracou field station, a segment of stem from each test approximately 2 cm in length was stored in 60% alcohol. In the laboratory, all segments were cut or sawn transversely, and the cut surface then cut or “shaved” cleanly with a fresh razor. The cut segment about 1 cm thick was then placed with its cut surface uppermost and flat (adjusted horizontally by pivoting the specimen on modelling clay until exactly horizontal) in a glass container and submerged in distilled water just covering, about 2 mm above the cut surface. The stem transverse section was then illuminated with fiber optic light source and photographed with an Olympus SZX9 binocular dissecting microscope (equipped with an Olympus DP72 digital camera). This preparation therefore produced the entire plant stem cross sections with good contrast between tissue types and without shrinkage or fragmentation of the section. A sub-sample of representative stems were paraffin embedded, microtome-sectioned, and stained using standard protocols. This sub-sample provided representative details of fine-scale histology.

Color images of stem sections were observed, and the principal tissue areas were delimited manually by tracing tissue areas using a fine fiber-tip pen on tracing paper. Tracings were then digitized and the cross-sectional areas (mm²) and the second moment of areas (mm^4^) of each tissue area measured using the image analysis software Optimas V.6.5.172, Media Cybernetics, Inc, Rockville, MD, USA, *via* thresholding of the manually drawn outlines and using a macro for second moments of area (courtesy of T. Almeras, Montpellier).

**Figure 3:**
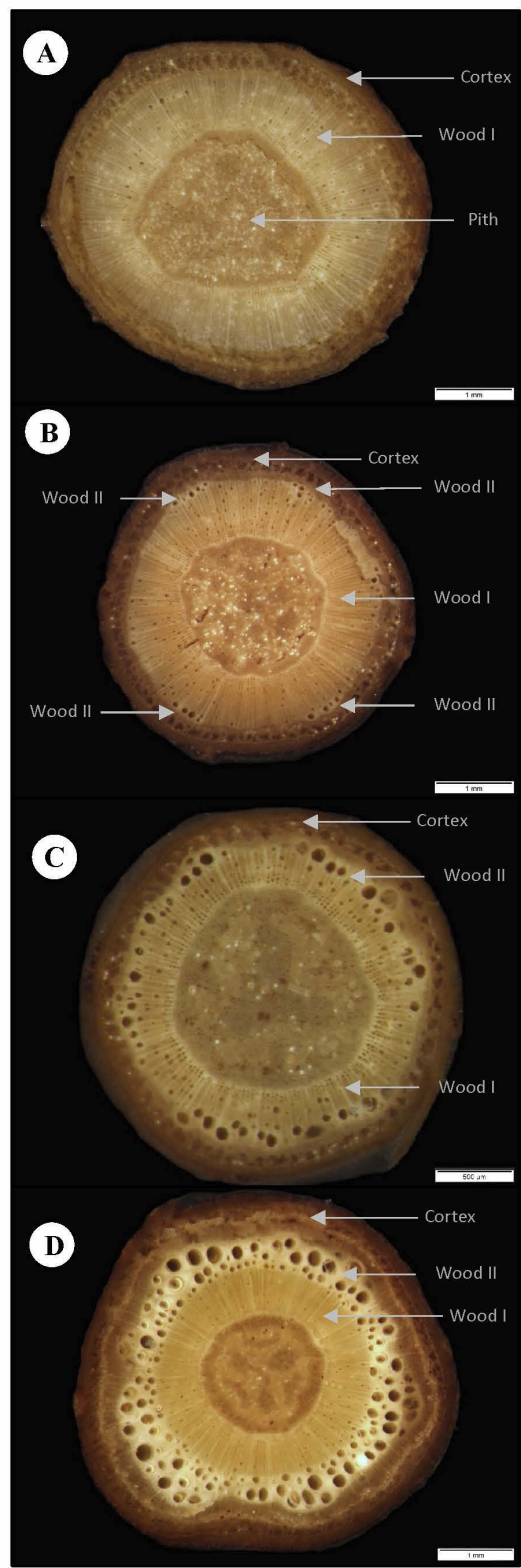
Representative cross-sectional tissue configurations of the four stem categories constituting the trellis systems of *C guianense*. **(A)** Self-supporting: A central pith is surrounded by a band of dense TYPE I wood which is surrounded by the “cortex”, which here includes the primary and secondary phloem and the primary and secondary cortical tissues. **(B)** Pendulous: Same organization as in **(A)**. In some basal pendulous axes, small pockets of secondary wood that resemble TYPE II wood and which can develop at the periphery of the cylinder of TYPE I wood (arrows). **(C)** Climbing: This section represents the level of development of TYPE II wood just below the twining attachment (note the narrow diameter of this segment). The cylinder of TYPE I wood is only narrow, and the layer of TYPE II wood with large vessels and soft interstitial tissue is nearly continuous. **(D)** Fixed: This section represents part of a fixed stem near the base of the fixed segment. Before attachment, the early stage of development as a young self-supporting stem had developed a wide cylinder of TYPE I wood. Following attachment an outer band of TYPE II wood has now developed with large vessels and soft interstitial tissue. Both the secondary phloem and periderm are well developed.

Tissue areas were generalized (**Figure 3**): (i) the central pith area (**Figure 3A**); (ii) the inner band of juvenile dense wood typical of the self-supporting phase of growth (**Figure 3A**); (iii) the later-formed area of mature less dense (lianoid) wood; in the early stages of climbing stages and in pendulous stems, this was initially formed as discontinuous lobes around the juvenile wood (**Figure 3B**) before becoming an entire ring-like layer (**Figures 3C, D**); (iv) the outer cortex of predominantly soft tissue that included the secondary and remaining primary phloem, small areas of latex canals, some small areas of lignified fibers, and periderm tissue (**Figure 3D**).

### Statistical analysis

Analyses of liana stems from natural habitats are well known to be difficult because of their pronounced indeterminate growth, their haphazard distribution, branching and clonality, and, particularly in this study, their development and “behavior” that can morph their structural organization from pre-attached to attached stems and many variants between. All data were analyzed using non-parametric Kruskal–Wallis (KW) tests followed by Dunn’s *post-hoc* tests. From a biological point of view, non-parametric comparisons are more suitable for comparisons of stem properties that vary along different stem lengths and between growth modes. We elected to conduct non-parametric comparisons and *post-hoc* tests of pooled stem diameter classes into three ranges of stem diameter: D_Narrow_, 1.8–3.9 mm; D_Medium_, 3.9–5.9; D_Large_, > 5.9 for each of the four types of growth. Our comparisons therefore compare similar stem diameters between different trellis growth modes. Because pith size and primary body diameter did not vary greatly between growth modes (see Results), stem diameter largely varied *via* the amounts of secondary growth rather than initial primary body size. We therefore decided to pool the data into diameter classes following the approach in (Ménard et al., 2009; Ménard et al., 2013; Soffiatti and Rowe, 2020) based on their variable amounts of secondary growth. Tissue correlations in terms of proportions of TYPE I and II wood to the second moment of area of the stem and Young’s modulus were carried out using non-parametric Spearman rank-order correlation coefficients.

## Results

### Trellis structure

The forest margin contained a highly complex diversity of young liana stems of *C. guianense*, closely intermingled and connected with self-supporting trees. Upright stems of the liana were highly noticeable emerging from the liana-tree trellis (**Figure 2A**). Pendulous stems were commonly visible on the forest margin suspended from their last point of attachment in the trellis. Self-supporting and pendulous stems bear leaves and this is especially marked in pendulous stems (**Figure 2B**). Both climbing and fixed stems occurred within patches of trellis up to about a meter inside the forest margin. Climbing stems, showing one or more turns around a host support, were also leafy (**Figure 2C**). Fixed stems, with two points of attachment, were more common as older stems within the trellis (**Figure 2D**). The trellis organization was therefore highly complex in terms of liana stem orientation and behavior for shoots having a similar stem diameter class of 1–10 mm in diameter.

### Mechanical properties of the trellis

#### Growth in diameter

**Figure 4:**
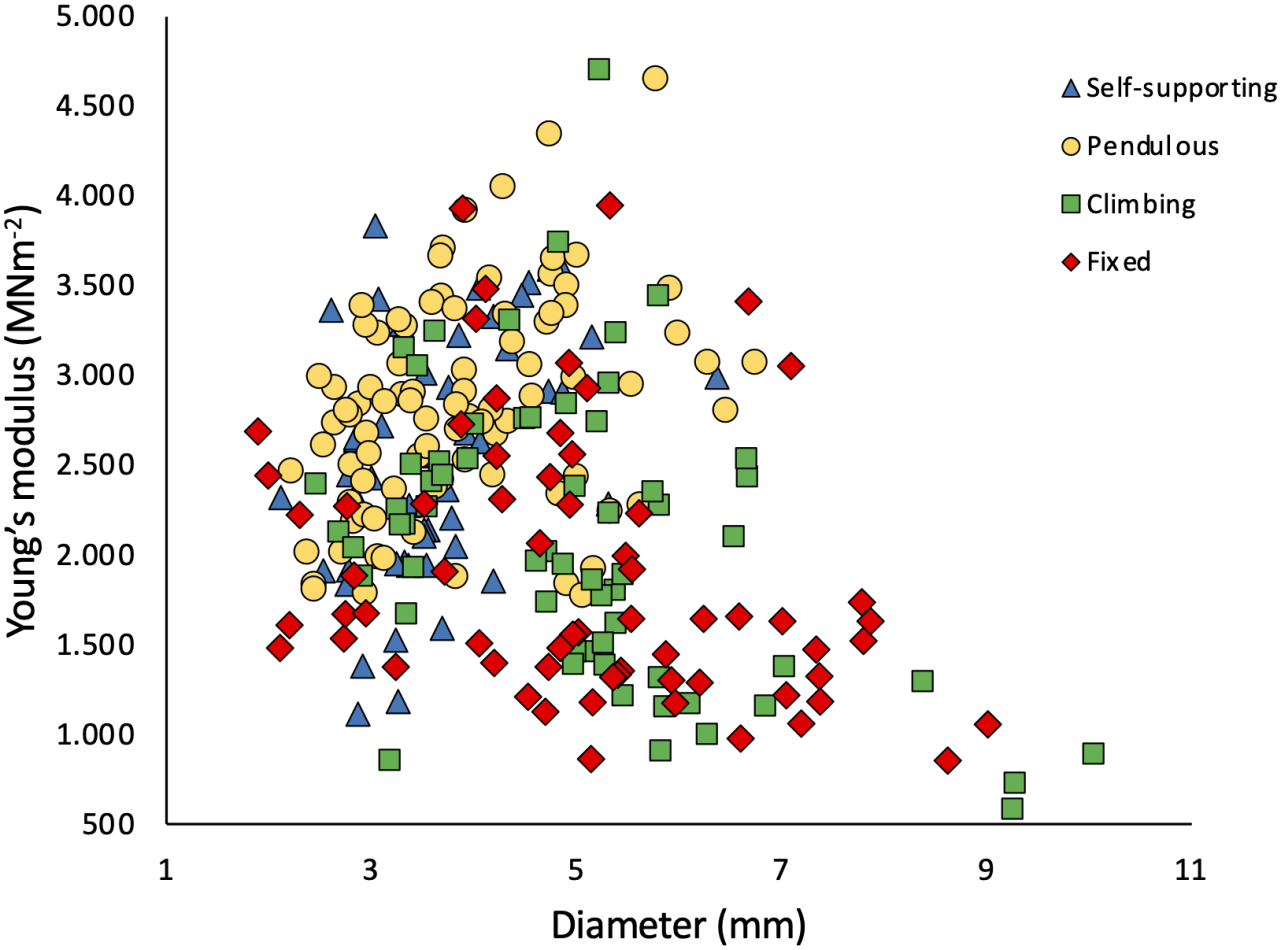
Bivariate scatter plot of trellis stem diameters and Young’s modulus of the stem measured in three-point bending. The stems tested include four categories: self-supporting, pendulous, climbing, and fixed. The data indicate that all kinds of growth habit can exist as narrow diameter branches, which increase in stiffness with increasing diameter. After a threshold diameter of 5–6 mm, only attached stems continue to grow and show a reduced stem stiffness.

Stem diameter across all four categories of shoot varied between approximately 2 and 10 mm (**Figure 3**). Mean stem diameters only varied significantly among stem types (**Table 1**). Climbing stems reached the highest mean values (5.11 ± 0.50 mm), whereas self-supporting and pendulous stems had the smallest mean values (4.50 ± 0.42 mm and 4.7 ± 0.54 mm, respectively; **Table 1**). The plot of stem diameter against stem stiffness (**Figure 4**) indicates that growth categories can exist as small diameter stems including self-supporting, pendulous, twining, and fixed shoots.

**Table 1:** Morphological and mechanical traits (means and SD) of four stem types (self-supporting, pendulous, climbing, and fixed) of *Condylocarpon guianense* categorized in function of diameter interval classes with *P*-values of Kruskal–Wallis and *post-hoc* Dunn’s test; small letters indicate significant differences.

Diameter	Narrow (1.8–3.9 mm)				
Traits/Stem types	Self-supporting	Pendulous	Climbing	Fixed	*P*-values
*Diameter CS (mm)*	3.22 ± 0.43^a^	3.15 ± 0.46^a^	3.27 ± 0.35^a^	2.85 ± 0.68^a^	p = 0.15
*Area CS (mm^2^)*	8.20 ± 2.28^a^	7.94 ± 2.27^a^	8.47 ± 1.70^a^	6.70 ± 3.12^a^	p = 0.1744
*% area of tissues CS*								
*Cortex*	33.36 ± 3.98^a^	31.95 ± 4.42^b^	36.02 ± 4.19^c^	36.45 ± 3.65^c^	p < 0.0001
*Wood Type I*	32.35 ± 8.62^a^	38.92 ± 8.55^b^	26.35 ± 10.46^ac^	22.57 ± 9.59^c^	p < 0.0001
*Wood Type II*	0.67 ± 2.24^a^	1.39 ± 2.89^a^	10.17 ± 8.96^b^	12.54 ± 8.31^b^	p < 0.0001
*Pith*	33.62 ± 8.42^a^	27.75 ± 6.21^b^	27.45 ± 8.45^b^	28.44 ± 8.76^ab^	p < 0.05
*Mechanical traits*								
*I (mm^4^)*	5.53 ± 2.92^a^	5.46 ± 3.02^a^	6.19 ± 2.90^a^	4.74 ± 4.36^a^	p = 0.1289
*E (MNm^−2^)*	2,332.20 ± 636.62^a^	2,704.18 ± 528.83^b^	2,286.09 ± 555.64^a^	2,113.88 ± 665.52^a^	p < 0.001
*EI (Nmm^2^)*	13,855.89 ± 8,070.42^a^	15,406.89 ± 10,160.33^a^	14.167.14 ± 7014.71^a^	10,306.44 ± 12,414.99 ^b^	p < 0.05
*Contributions to I (%)*								
*Cortex*	55.2 7 ± 5.14^a^	53.11 ± 4.65^b^	57.82 ± 3.58^c^	58.88 ± 4.71^c^	p < 0.0001
*Wood Type I*	31.78 ± 7.50^a^	37.03 ± 7.93^b^	22.29 ± 9.13^c^	18.42 ± 8.54^c^	p < 0.0001
*Wood Type II*	0.75 ± 2.55^a^	1.61 ± 3.35^b^	11.33 ± 9.29^c^	13.80 ± 8.35^c^	p < 0.0001
*Pith*	12.19 ± 5.95^a^	8.25 ± 3.42^b^	8.56 ± 4.81^b^	8.90 ± 4.98^ab^	p < 0.05

Diameter	Medium (3.9–5.9 mm)				
Traits/Stem types	Self-supporting	Pendulous	Climbing	Fixed	*P*-values
*Diameter CS (mm)*	4.50 ± 0.42^a^	4.7 ± 0.54^ac^	5.11 ± 0.50^b^	4.90 ± 0.52^bc^	p < 0.001
*Area CS (mm^2^)*	16.08 ± 3.03^a^	17.58 ± 4.08^ac^	20.67 ± 3.92^b^	18.60 ± 3.78^bc^	p < 0.001
*% area of tissues CS*								
*Cortex*	31.12 ± 4.10^a^	33.64 ± 6.01^a^	37.22 ± 4.81^b^	38.98 ± 4.53^B^	p < 0.00001
*Wood Type I*	47.53 ± 7.28^a^	43.89 ± 9.56^a^	24.64 ± 13.03^b^	23.06 ± 11.79^b^	p < 0.0001
*Wood Type II*	0.07 ± 0.25^a^	2.66 ± 5.07^a^	22.96 ± 12.93^b^	23.76 ± 12.18^b^	p < 0.0001
*Pith*	21.29 ± 4.84^a^	19.81 ± 5.84^a^	15.19 ± 5.4^b^	14.20 ± 4.68^b^	p < 0.0001
*Mechanical traits*								
*I (mm^4^)*	20.63 ± 8.96^a^	24.09 ± 13.58^ac^	32.42 ± 11.55^b^	27.78 ± 10.01^bc^	p < 0.05
*E (MNm^−2^)*	3,027.49 ± 513.35^a^	3,015.03 ± 682.70^a^	2,221.55 ± 844.77^b^	2,032.64 ± 790.35^b^	p < 0.00001
*EI (Nmm^2^)*	65,077.09 ± 25,845.67^a^	78,503.71 ± 45,766.80^a^	75,175.21 ± 39,016.44^a^	56,413.41 ± 26,912.42^a^	p = 0.0637
*Contributions to I (%)*								
*Cortex*	52.31 ± 5.70^a^	55.05 ± 6.43^a^	59.83 ± 5.86^b^	62.07 ± 5.17^b^	p < 0001
*Wood Type I*	42.77 ± 6.83^a^	37.68 ± 10.12^a^	16.11 ± 13.15^b^	14.11 ± 10.47^b^	p < 0.0001
*Wood Type II*	0.08 ± 0.32^a^	2.60 ± 5.21^a^	21.37 ± 10.84^b^	21.33 ± 8.79^b^	p < 0.0001	*Pith*	4.83 ± 2.20^a^	4.67 ± 3.75^a^	2.69 ± 1.78^b^	2.49 ± 1.52^b^	p < 0.0001

Diameter	Large (>5.9 mm)				
Traits/Stem types	Self-supporting	Pendulous	Climbing	Fixed	*P*-values
*Diameter CS (mm)*	6.36 ± 0.00^na^	6.4 ± 0.31^a^	7.5 ± 1.4^a^	7.1 ± 0.8^a^	p = 0.2226
*Area CS (mm^2^)*	31.80 ± 0.00^na^	31.8 ± 3.12^a^	46.1 ± 17.7^a^	39.3 ± 10.9^a^	p = 0.3613
*% area of tissues CS*								
*Cortex*	36.1 ± 0.00^na^	30.2 ± 6.06^a^	42.0 ± 5.2^b^	40.7 ± 4.6^b^	p < 0.05
*Wood Type I*	49.8 ± 0.00^na^	51.6 ± 4.09^a^	16.0 ± 12.6^b^	19.81 ± 8.6**^b^**	p < 0.01
*Wood Type II*	0.0	1.6 ± 3.28^a^	33.2 ± 10.9^b^	30.4 ± 10.1^b^	p < 0.01
*Pith*	14.1 ± 0.00^na^	16.5 ± 5.4^a^	8.8 ± 3.5^b^	9.7 ± 3.1^b^	p < 0.05
*Mechanical traits*								
*I (mm^4^)*	76.1 ± 0.00^na^	70.9 ± 13.63^a^	165.3 ± 118.9^a^	127.0 ± 69.8^a^	p = 0.2161
*E (MNm^−2^)*	2,989.8 ± 0.00^na^	2,373.8 ± 812.79^a^	1,392.0 ± 671.3^b^	1,536.4 ± 652.6^b^	p < 0.01
*EI (Nmm^2^)*	243,677.2 ± 0.00^na^	180,835.9 ± 42,811.42^a^	21,7469.5 ± 113,480.3^a^	193,876 ± 100,307.0^a^	p = 0.4816
*Contributions to I (%)*								
*Cortex*	59.1 ± 0.00^na^	50.9 ± 8.15^a^	65.7 ± 6.0^b^	63.05 ± 3.4^b^	p < 0.05
*Wood Type I*	38.8 ± 0.00^na^	44.2 ± 10.07^a^	7.8 ± 9.8^b^	8.8 ± 6.3^b^	p < 0.01
*Wood Type II*	0.0	1.8 ± 3.59^a^	25.5 ± 6.0^b^	26.4 ± 7.4^b^	p < 0.01
*Pith*	2.0 ± 0.00^na^	3.1 ± 1.49^a^	1.1 ± 0.7^b^	1.3 ± 0.6^b^	p < 0.05

Kruskal–Wallis test was not performed including self-supporting samples on diameter class D_Large_ due to limited sample size.

Above a diameter of 5–6 mm, there were very few self-supporting or pendulous axes. Above this threshold, only attached, climbing (single attachment point), or fixed (two attachment points) develop larger diameters (**Figure 4**). Furthermore, these larger attached axes also tend to show lower stiffness with values of Young’s modulus below approximately 2,500 MPa (**Figure 4**). Overall, Young’s modulus increases with stem diameter up to a diameter of approximately 5–6 mm. After this, only trellis segments that are attached develop significantly more in diameter and show a decrease in Young’s modulus.

#### Changes in stem stiffness (Young’s modulus)

**Figure 5:**
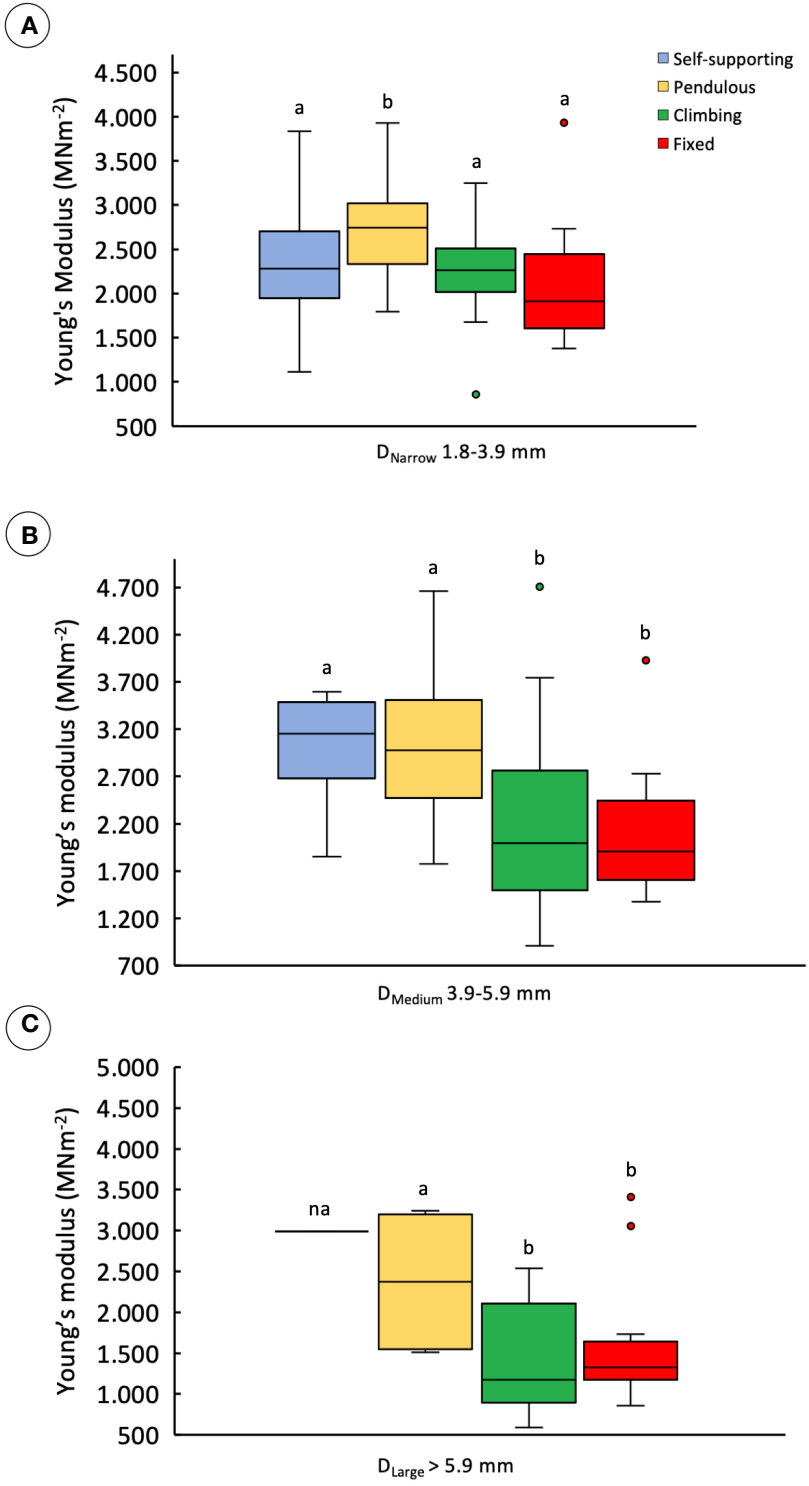
Young’s modulus of stem categories making up trellis grouped into three diameter classes: **(A)** D_Narrow_, 1.8–3.9 mm; **(B)** D_Medium_, 3.9–5.9 mm; **(C)** D_Large_, >5.9 (inner lines, medians; boxes, 25th and 75th percentiles; whiskers, max and min values; excluding outliers, circles extreme values). Small letters indicate significant differences (Dunn’s *post-hoc* test) between median values of stem types.

Comparisons of stem stiffness between stem categories for the same diameter (**Table 1**) indicated a slight difference in median Young’s modulus only for pendulous stems. These showed higher values than the other types in the D_Narrow_ category (**Figure 5A**) (KW, *p-*value < 0.001). For D_Medium_ stems (**Figure 5B**), both self-supporting and pendulous stem segments showed somewhat higher median stiffness than attached stems of the trellis (KW, *p-*value < 0.00001). Finally, among D_Larger_ stems, the Young’s modulus of all non–self-supporting forms showed lower stiffness, but with the unattached pendulous stems still showing higher stiffness than the attached stems (**Figure 5C**) (KW, *p-*value < 0.01).

### Tissue development

#### Types of wood

**Figure 6:**
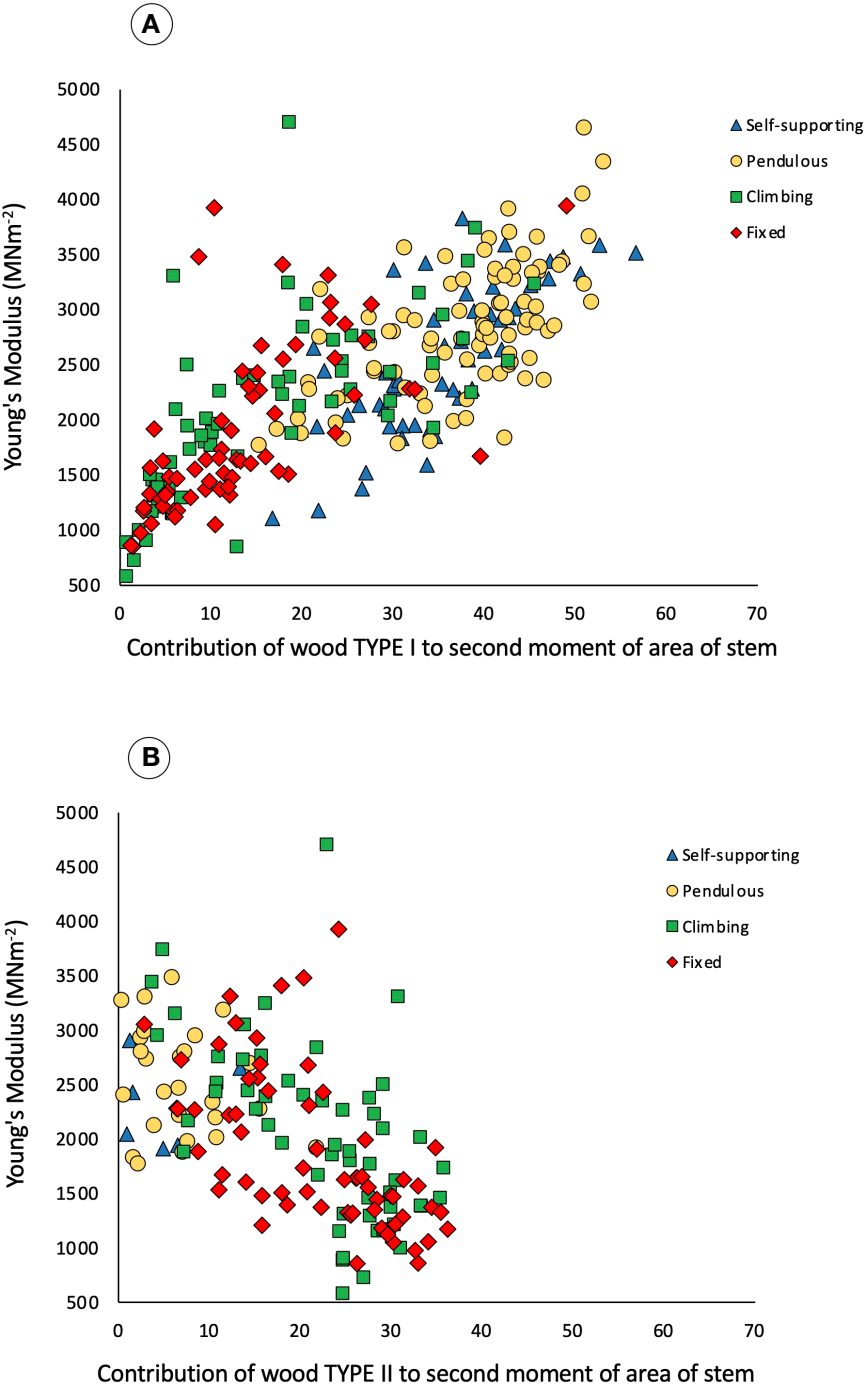
Bivariate scatter plot of trellis stem contribution of wood types to the second moment of area (%) and Young’s modulus of the stems measured in three-point bending of the four categories: self-supporting, pendulous, climbing, and fixed. **(A)** Contribution of wood TYPE I to the second moment of area and Young’s modulus. Spearman rank-order correlation (n = 269), R_s_ = 0.7503 (t = 18.54), P < 0.001. Climbing and fixed stems show a lower contribution as they only develop Type I wood at the beginning of their growth and can also produce significant amounts of Type II wood. **(B)** Contribution of wood TYPE II to the second moment of area and Young’s modulus, Spearman rank-order correlation (n = 269), R_s_ = −0.5720 (t = −11.40), P < 0.001. Self-supporting and pendulous stems have a little contribution as they barely develop lianoid wood.

Initially, all stem categories have the same kind of wood, termed TYPE I. This is composed of apparently denser fiber tissues (visible as thicker cell walls and smaller lumens) and of narrow vessel elements in the predominantly fibrous wood tissue (**Figure 3A**). Wood TYPE II is formed around the periphery of TYPE I wood and is composed of larger vessels, embedded in patches of less dense, tissue typical of lianoid wood, here called wood TYPE II (**Figures 3B–D**). Across all growth categories, Young’s modulus increases with increasing percentage of wood TYPE I contribution to the second moment of area of stems (**Figure 6A**). The opposite was observed for the percentage of area of wood TYPE II (**Figure 6B** and **Table 1**). It starts to be produced after TYPE I, especially in climbing and fixed stems, where it will be the principal component of the woody cylinder.

#### Changes of wood type with stem behavior in the trellis

**Figure 7:**
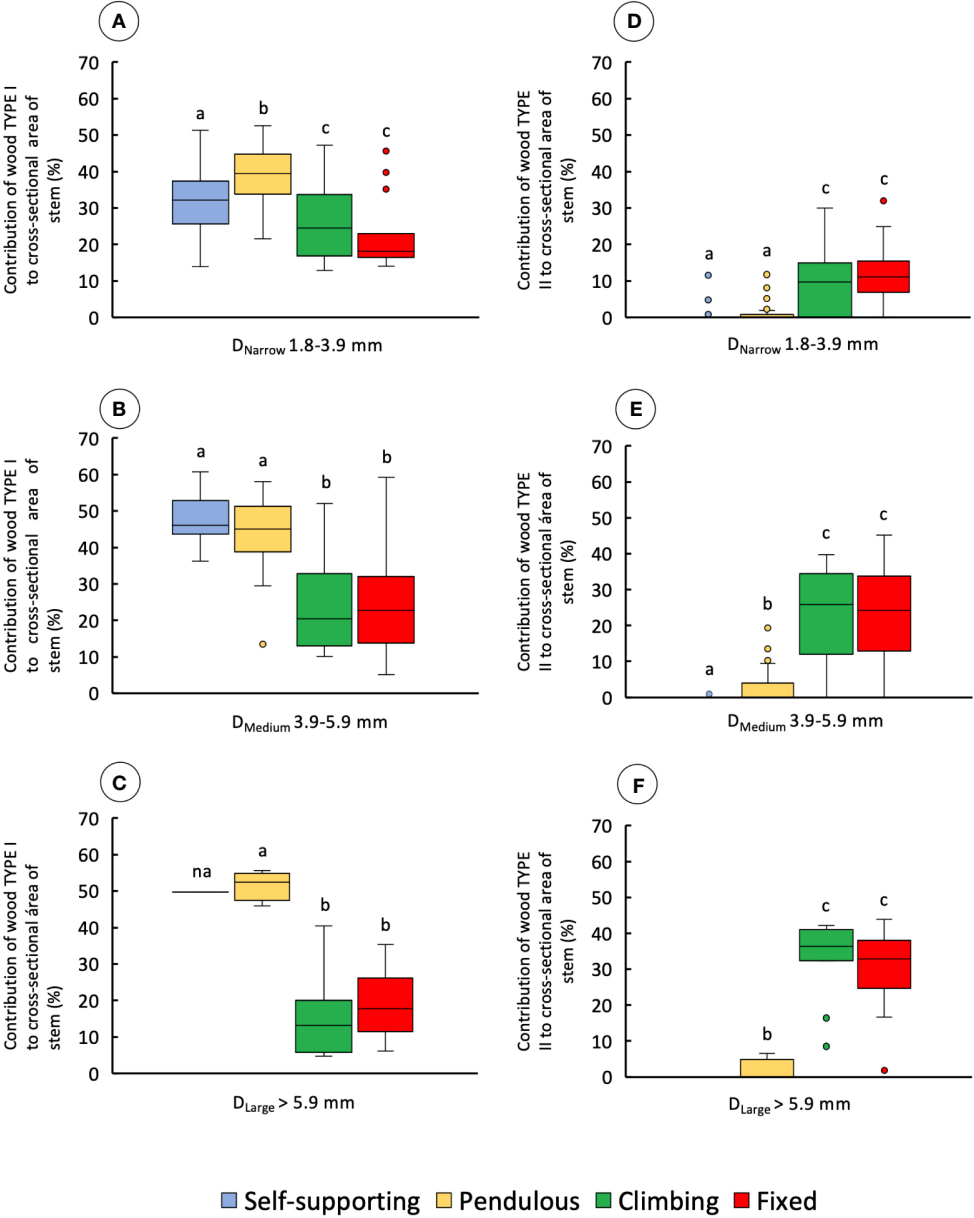
Percentage contribution of wood types to cross-sectional area for the four stem types: self-supporting, pendulous, climbing, and fixed, by classes of diameter (D_Narrow_, D_Medium_, and D_Large_). **(A–C)** contribution of wood TYPE I to the cross-sectional area of stems. **(D–F)** Contribution of wood TYPE II to the cross-sectional area of stems (inner lines, medians; boxes, 25th and 75th percentiles; whiskers, max and min values; excluding outliers, circles extreme values). Small letters indicate significant differences (Dunn’s *post-hoc* test) between median values of stem types.

Cross-sectional area of wood TYPE I varied significantly among stem types in all diameter classes (**Figures 7A–C** and **Table 1**). Wood TYPE I is proportionally more abundant in D_Narrow_ and D_Medium_ stems, especially in self-supporting and pendulous shoots (KW, *p-*values < 0.0001 and 0.01, respectively). Pendulous stems had the highest mean values in D_Narrow_. Self-supporting and pendulous stems showed approximately twice that of climbing and fixed stems. In D_Large_, it was approximately three times higher than that in climbing and fixed stems (KW, *p-*values < 0.01).

Despite significant differences among stem types in all diameter classes (**Figures 7D–F**), climbing and fixed shoots had the highest mean values for wood TYPE II. The great majority of self-supporting and pendulous stems showed an absence of wood TYPE II apart from some older buckled pendulous stem sections (**Table 1**).

In self-supporting and pendulous stems, the proportion of TYPE 1 wood to the second moment of area of the stem was higher than that in climbing and fixed stems in all diameter classes (**Table 1**). The relative proportion of TYPE II wood to the second moment of area was significantly higher in climbing and fixed stems (**Table 1**).

#### Pith and cortex

**Figure 8:**
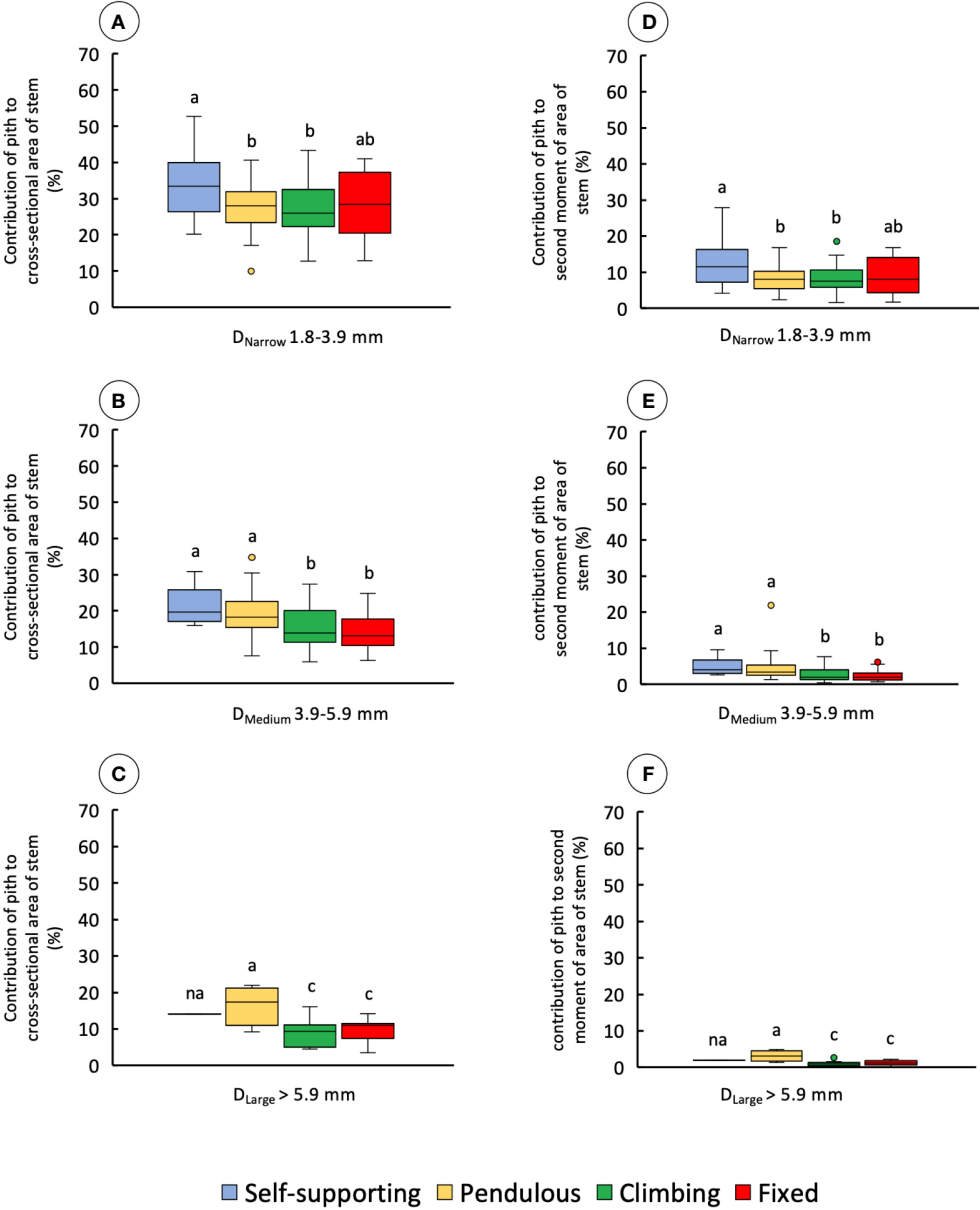
Percentage of contribution of pith to the cross-sectional area and second moment of area of the four stem types: self-supporting, pendulous, climbing, and fixed, by classes of diameter (D_Narrow_, D_Medium_, and D_Large_). **(A–C)** Contribution of pith to the cross-sectional area of stems. **(D–F)** Contribution of pith to the second moment of area of stems (inner lines, medians; boxes, 25th and 75th percentiles; whiskers, max and min values; excluding outliers, circles extreme values). Small letters indicate significant differences (Dunn’s *post-hoc* test) between median values of stem types.

The proportion of pith to the cross-sectional area of the stem varied significantly among diameter classes and stem categories (**Figures 8A–C** and **Table 1**).

Among narrow stems, D_Narrow_, self-supporting stems had proportionally larger areas of pith, whereas other stem types varied somewhat but with no significant differences (KW, *p-*values < 0.05). Among medium-diameter stems, self-supporting and pendulous stems had the largest proportions of pith area (KW, *p-*values < 0.0001). Among broad stems, pendulous stems also showed the highest proportions of pith tissue (KW, *p-*values < 0.05) although few stems of this category reached this diameter. These values were also mirrored in terms of the second moment of area (**Figures 8D–F** and **Table 1**) among narrow stems (KW, *p-*values < 0.05), medium stems (KW, *p-*values < 0.0001), and broad stems (KW, *p-*values < 0.05).

**Figure 9:**
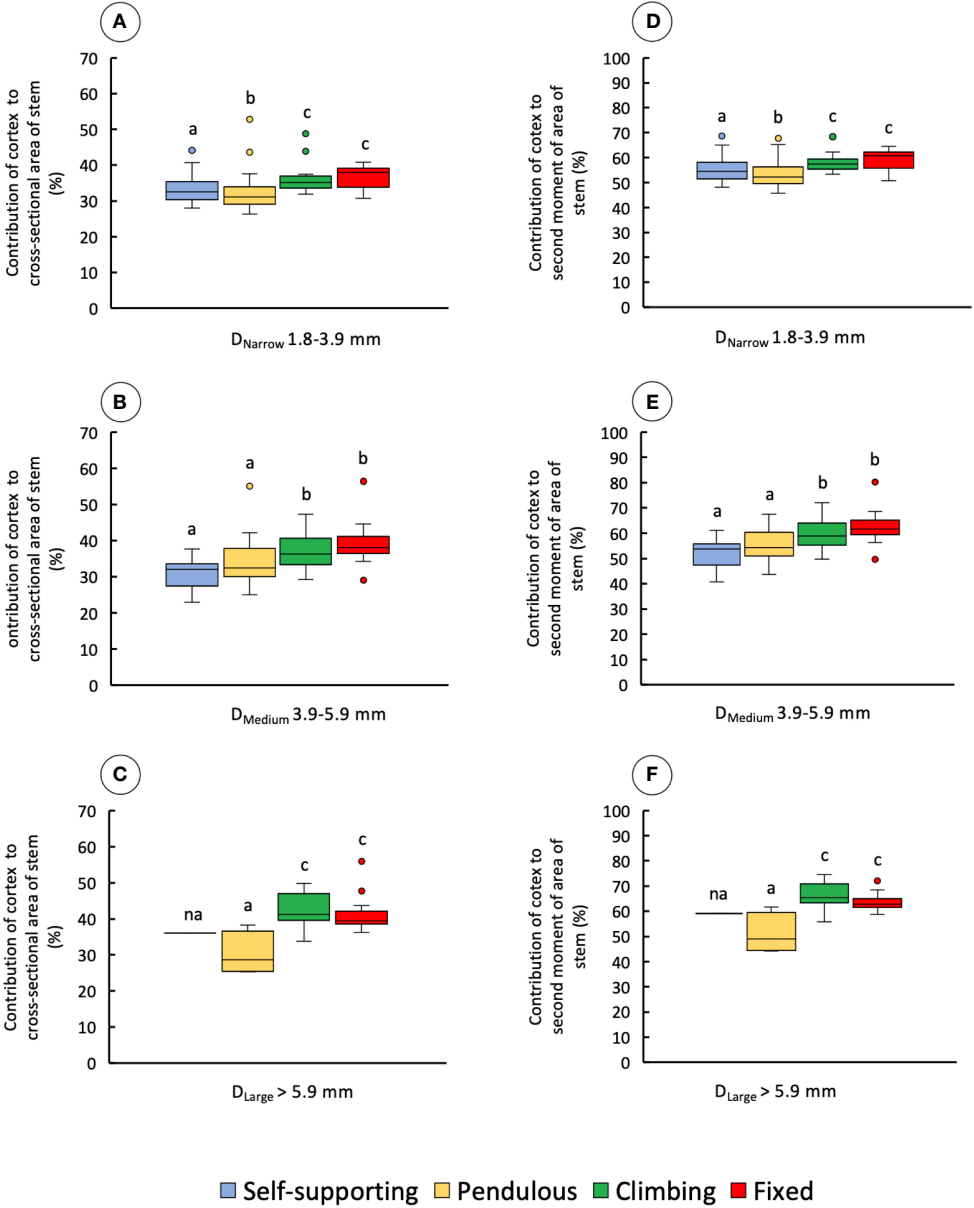
Percentage of contribution of cortex to the cross-sectional area and second moment of area of the four stem types: self-supporting, pendulous, climbing, and fixed, by classes of diameter (D_Narrow_, D_Medium_, and D_Large_). **(A–C)** Contribution of cortex to the cross-sectional area of stems. **(D–F)** Contribution of cortex to the second moment of area of stems (inner lines, medians; boxes, 25th and 75th percentiles; whiskers, max and min values; excluding outliers, circles extreme values). Small letters indicate significant differences (Dunn’s *post-hoc* test) between median values of stem types.

The proportion of cortex to the cross-sectional area of the stem also varied significantly among stem samples and diameter classes (**Figures 9A–C** and **Table 1**). In narrow stems, median values were different among all stem types, with climbing and fixed stems having higher mean values than pendulous and self-supporting ones (KW, *p-*values < 0.0001). In medium-diameter stems, self-supporting and pendulous axes had lower values than the other two stem types (KW, *p-*values < 0.00001). The same was observed for large diameters in which pendulous stems had the lowest areas of cortex (KW, *p-*values < 0.05). Climbing and fixed stems had higher contributions of cortex to the second moment of area of the stem than the other stem categories (**Figures 9D–F** and **Table 1**).

## Discussion

Liana trellises are a widespread and ecologically important structural feature in tropical humid forests. They allow young stages of development of lianas to remain aloft in dense canopy and permit invasion and colonizing of new spaces. They are also an essential structural feature as physical niches for many other arboreal organisms to position themselves or move through the forest habitats (Schnitzer et al., 2015). However, the underlying organization, the adaptive shifts in mechanical properties, and the growth in constructing such structures remain understudied. One of the principal difficulties in studying trellis organization in the field rather than experimentally observed setups is their sheer haphazard complexity (**Figure 1**) and the difficulty of sampling discrete individuals and growth units.

A key adaptive feature of liana trellises is that they permit a safe, secure attachment and a mechanically robust net-like system that maintains an aerial position in and between trees. Because lianas do not develop massive self-supporting trunks, their trellis systems (effectively their attached canopies) must be constructed by young shoots. The liana studied here makes up complex trellis systems *via* growth and development of young cauline stems. These have the same “starting point” (similar primary body size and organization of pith and cortex) and, as young shoots, develop to perform very different tasks. This study explored some of the morpho-anatomical transitions and mechanical properties underlying this “constructional phase” by young shoots. We characterized four of the principal growth behaviors that build up the liana trellis.

### Self-supporting stems (searching)

**Figure 10:**
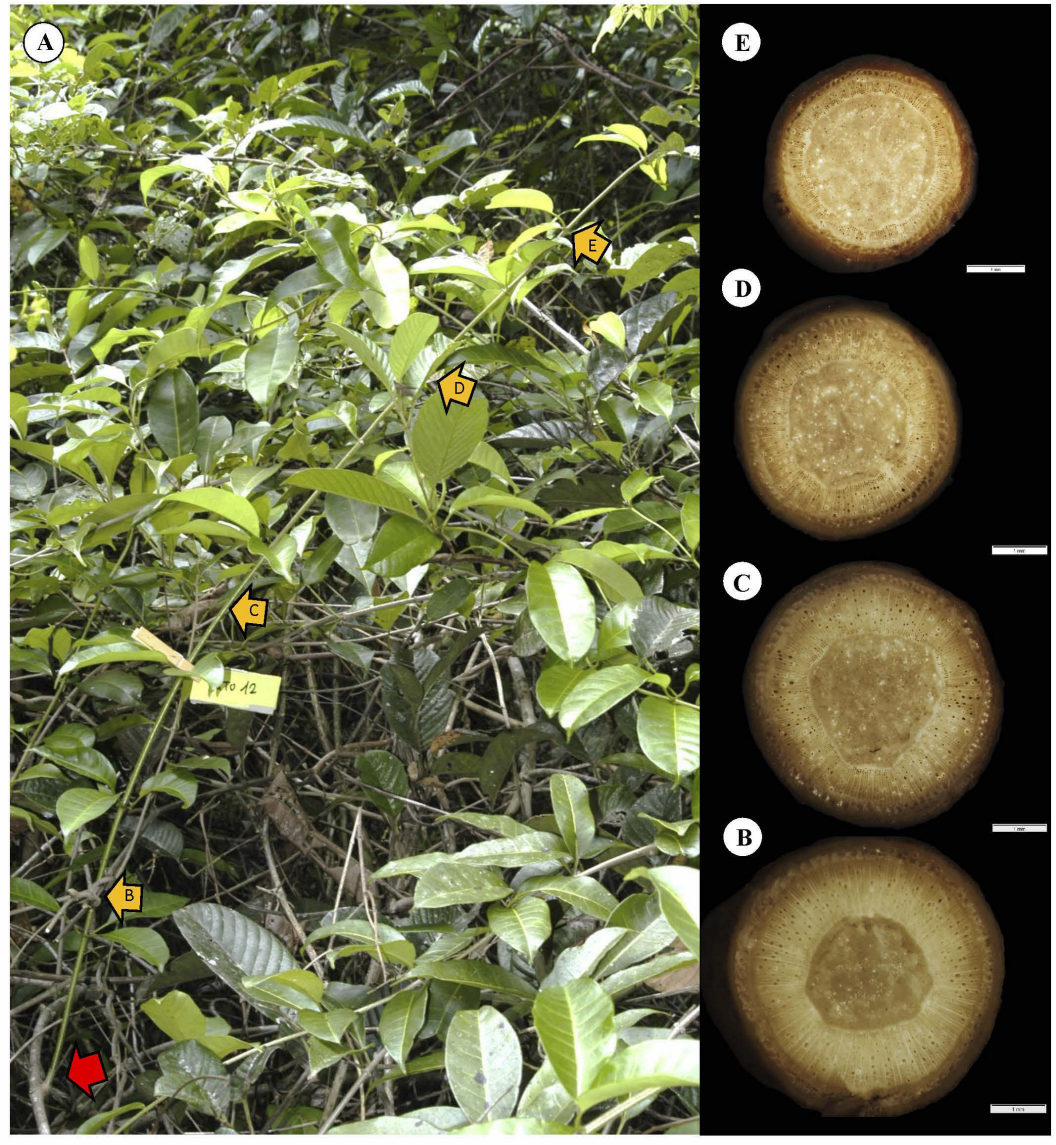
Representative self-supporting stem of *C guianense*. **(A)** In exact field position at time of collection along with macro-anatomical sections [**(B–E)** and yellow arrows] along the stem. The red arrow indicates the basal point of attachment of the self-supporting axis. The stem is in close proximity to neighboring trellis branches but has not made an attachment and still remains self-supporting (compare with **Figure 2A**, which extends and “searches” some distance from the trellis). The stem produced a cylinder of uniform, homogeneous stiff TYPE I wood with only small vessels (see Discussion).

To initiate trellis formation, supports must be located by crossing open spaces (**Figure 10**). The self-supporting axes concentrate resources as stem stiffness *via* dense TYPE I wood, ensuring reach. Most self-supporting searchers, as illustrated here, exhibit tapered wood cylinders. The apical most part of the shoot undergoes circumnutatory movements. The wood all along searcher stems is homogeneous with few small-diameter vessels from the base to the apex of the shoot (**Figures 10B–D**). A relatively wide pith acts as a mechanical spacer in these slender stems (Mahley et al., 2018), increasing the second moment of area and enhancing rigidity and presumably reach.

Our results indicated that none of the self-supporting stems produced dense stiff TYPE I wood in stems having a diameter greater than 5–6 mm in diameter. This suggests a hard-wired, cutoff point where stiff juvenile wood development is stopped. It appears that the trellis-forming capacity across gaps can only be formed at a primary distance of *c.* 1.5 m represented by searcher maximal length—the approximate length of self-supporting searchers in this species (Hattermann et al., 2022). There is no indeterminate or extended phase of support exploration as self-supporting stems. Instead, there appears to be limit of three-dimensional (3D) space for which this species is adapted to exploit. In comparison with other searcher lengths (Putz, 1984; Hattermann et al., 2022), different climbing plants have arguably undergone selection for specific habitats that include different distances between supports. In other words, at least for *C. guianense*, the plant does not invest materially in a self-supporting mode of growth for searching aerially further than that it was under selection for.

### Pendulous stems (resource production/light trapping)

**Figure 11:**
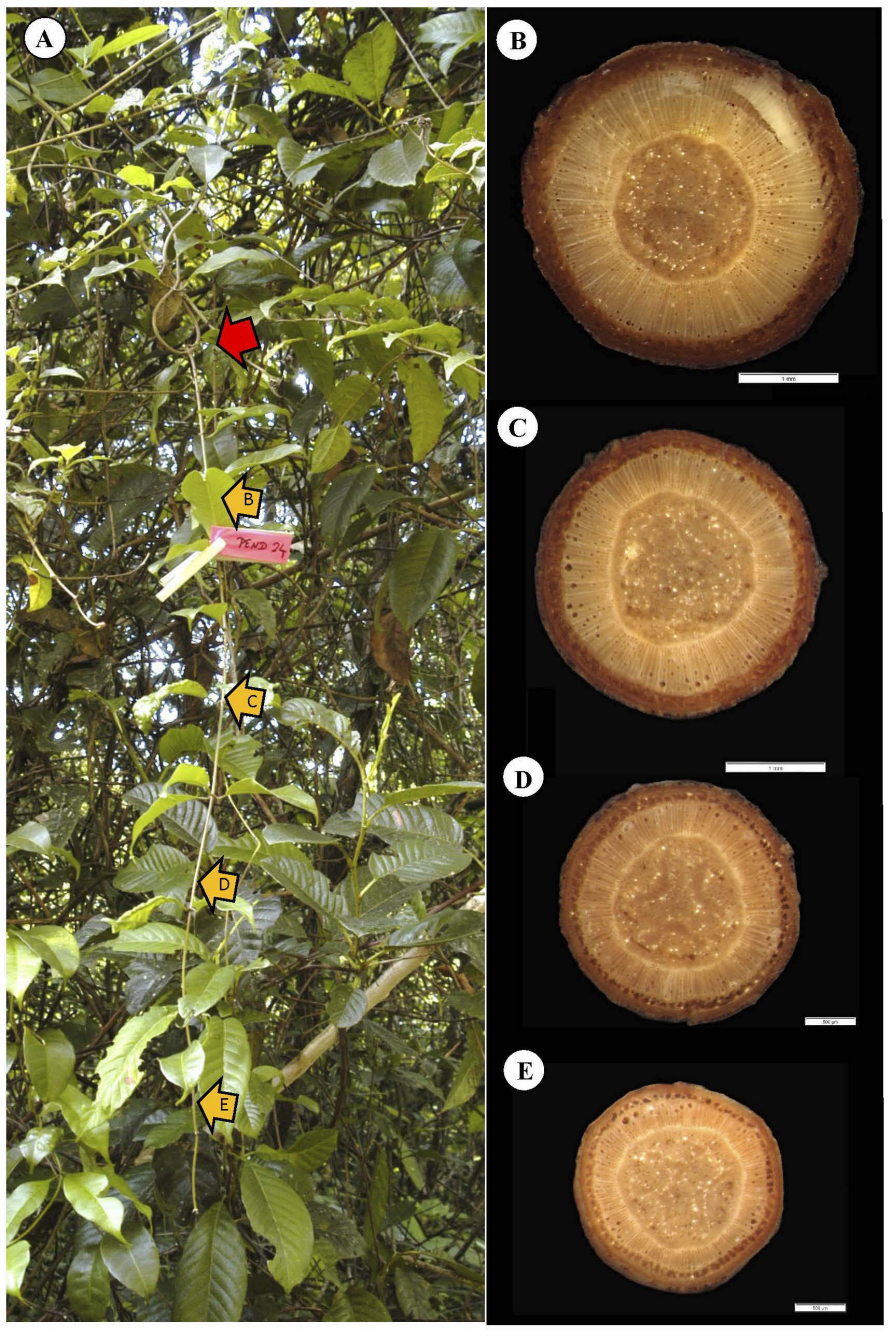
Representative pendulous stem of *C guianense*. **(A)** In exact field position at time of collection along with macro-anatomical sections [**(B–E)** and yellow arrows] along the stem. The red arrow indicates the point of attachment to a flexible, looped part of the liana trellis and from which point the originally self-supporting stem has deflected under its own weight. The stem presents numerous leaves and has developed medium to large vessels near the periphery of the TYPE I wood (see Discussion).

Self-supporting stems lose stability if they do not reach a support and switch to a pendulous orientation (**Figure 11A**). They show a similar anatomical organization as self-supporting stems (**Figures 11B–E**). They also stop producing TYPE I wood at a threshold of 5-6 mm stem diameter, and, if they produce any more wood, it is TYPE II wood with many large vessels and less dense surrounding tissue (**Figure 5E**). Many hanging stems retain leaves and remain in place presumably photosynthesizing and contributing photosynthate resources to the rest of the trellis. Some pendulous stems were observed to bend at their previous self-supporting base, others are attached to already flexible parts of the trellis, and it is this flexible connecting part of the trellis that deforms benignly (**Figure 11A**, red arrow). Furthermore, studies should examine how pendulous axes actually reconfigure and survive large bending and torsional strains when they lose stability. Some studies have demonstrated the resilience and toughness of older flexible liana stems, which can show remarkable patterns of stem compartmentalization following significant TYPE II wood development (Fisher and Ewers, 1989; Putz and Holbrook, 1991). However, not much is known about failure and toughness of young shoots prior to significant development of TYPE II lianoid wood in lianas. One study has related bending strength to fiber density in juvenile shoots prior to TYPE II wood development. It was found that young shoots and more mature shoots of wild-type *Manihot esculenta* showed ductile failure of the stem under three-point bending loads than more catastrophic brittle failure (Ménard et al., 2013). Future studies might explore how initial growth of TYPE II wood influences toughness and the type of failure in climbing stems as has been done for shrubs and trees (Ennos and van Casteren, 2010; van Casteren et al., 2012; Hone et al., 2021). Downward deflection of the stem and presence of large leaf areas is probably an important function in the formation of trellises where more searcher stems do not find a support than find them. The kind of “lateral blanketing” of trees and forest margins by pendulous trellis stems is prevalent, particularly in the tropics (**Figure 1**). Indeed, the mechanism that transitions from self-supporting to pendulous behavior in trellises *via* benign buckling is probably the widespread in vines and lianas, and it is possibly as much a mechanism in itself for light capture and provision of clonal trellis systems as it “fails” in connecting to a support.

### Climbing (connecting and transforming)

**Figure 12:**
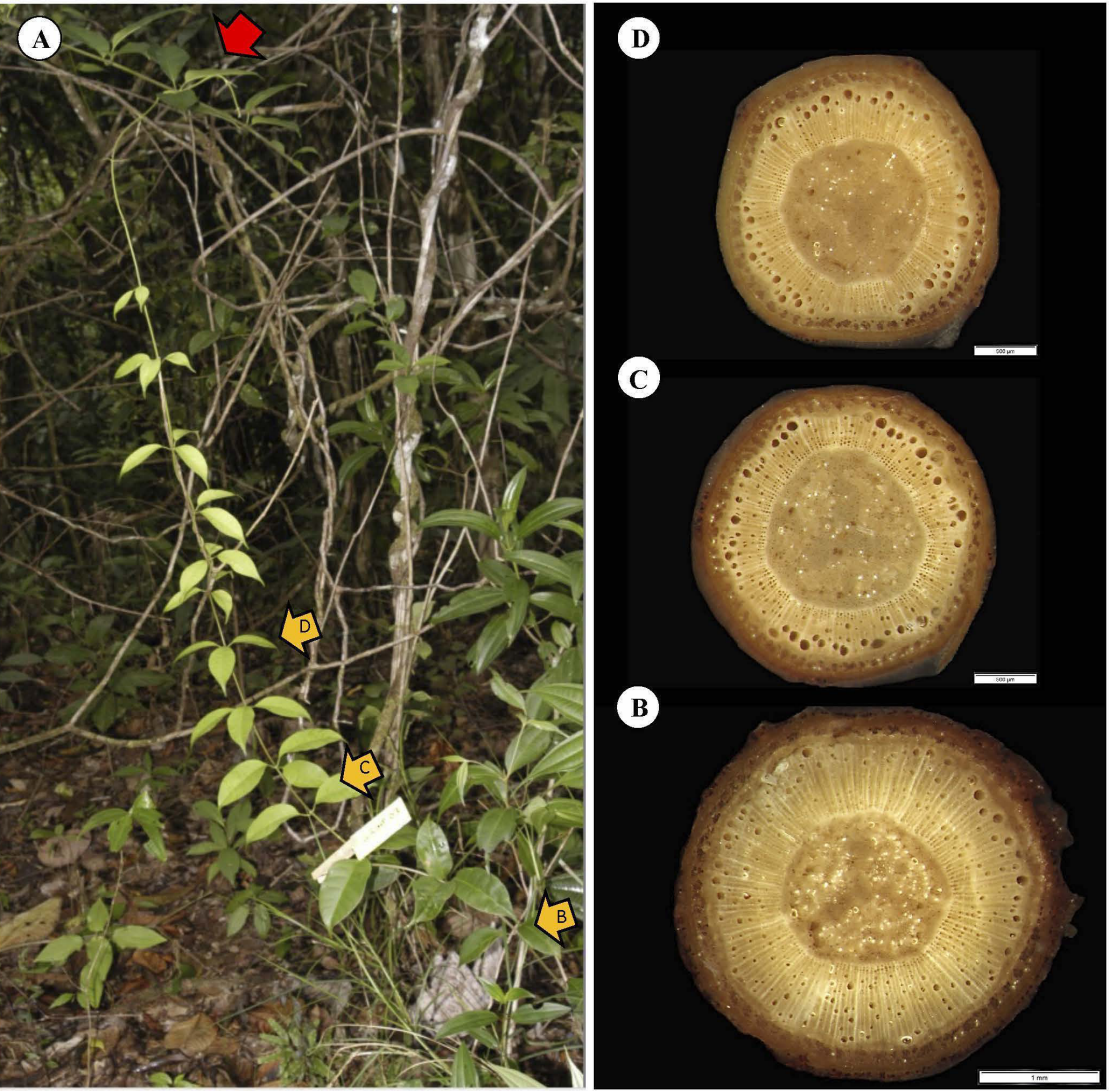
Representative climbing stem of *C guianense.* **(A)** In exact field position at time of collection along with macro-anatomical sections [**(B–D)** and yellow arrows] along the stem. The red arrow indicates a second climbing phase of the main stem where twining has failed to secure a fixed attachment to a leafy trellis stem of the same species and possibly the same clonal individual. The field position and anatomy indicate the exact moment at which TYPE II wood is produced by a climbing stem after twining around a support. At sections D and C, 5 and 15 cm below the first complete turn of the twined stem, respectively, TYPE II wood has developed around the periphery of the TYPE I wood cylinder. The basal most section **(B)** has developed medium to large vessels around the TYPE II wood periphery.

Self-supporting stems that attach *via* twining of a circumnutating stem apex (**Figure 2C**) represent the crucial connecting stage that builds up a liana trellis. Connection by twining of the main stem is probably one of the most wide spread modes of attachment in climbing plants (Gentry, 1991). It is also, arguably, one of the “simplest” attachment and climbing mechanisms. However, twining does require extra derived behavioral movements linked to growth. We show an example of attachment by twining in a representative stem *via* three turns around the host support (**Figure 12A**). TYPE II wood is produced approximately 5–10 cm below the lowest point of attachment (**Figure 12B**). At this point, localized areas of the cambium have produced approximately two layers of vessels with surrounding soft tissue that we term TYPE II wood. The connection event has also initiated the TYPE II wood development lower down the stem (**Figure 12C**), but, near the base of the stem, there is not much evidence of it apart from a few patches of wide vessels (**Figure 12D**). This lower part of the stem shows similar anatomy and mechanical properties as typical self-supporting stems of our data set. Above the point of attachment, the apical part of the climbing shoot had continued as a searcher in free space and then encountering a leafy shoot of the same trellis and the same species. At this point, it is hindered by an obstacle and unable to twine tightly (Lehnebach et al., 2022) and roves loosely across some branches without actually twining (**Figure 12A**, red arrow). This example illustrates that twining has initiated the TYPE II lianoid wood at a very early stage of development with only a radial diameter of 200 µm thickness of stiff wood (**Figure 12B**) and just below the actual phase of twining. The twining phase has initiated the development of TYPE II, lianoid wood, and a drop in Young’s modulus of the stem, initiating the flexible tissue composed of larger vessels, embedded in patches of less dense tissue that protects slender trellis stems from damage *via* mechanical loads.

Hence, during the formation of the trellis, the “connection” phase interrupts production of stiff wood very close to the point of contact when the twining stem is still young. Crucially, the connection also initiates and triggers the production of TYPE II wood down and along the stem effectively sheathing already formed TYPE I wood. There seems to be a lag between the initiation of TYPE II wood close to the point of attachment compared with further down the now attached climbing stem. The switch from TYPE I to TYPE II wood is now initiated along the searcher. Effectively, what was once composed of stiff tissues adapted to cross gaps has now become part of a flexible net-like trellis system (visible all around the young climbing shoot in **Figure 12A**), which has added another connection to the trellis and likely increased the safety and protection of the trellis as a whole from failure and falling from the supporting branches.

**Figure 13:**
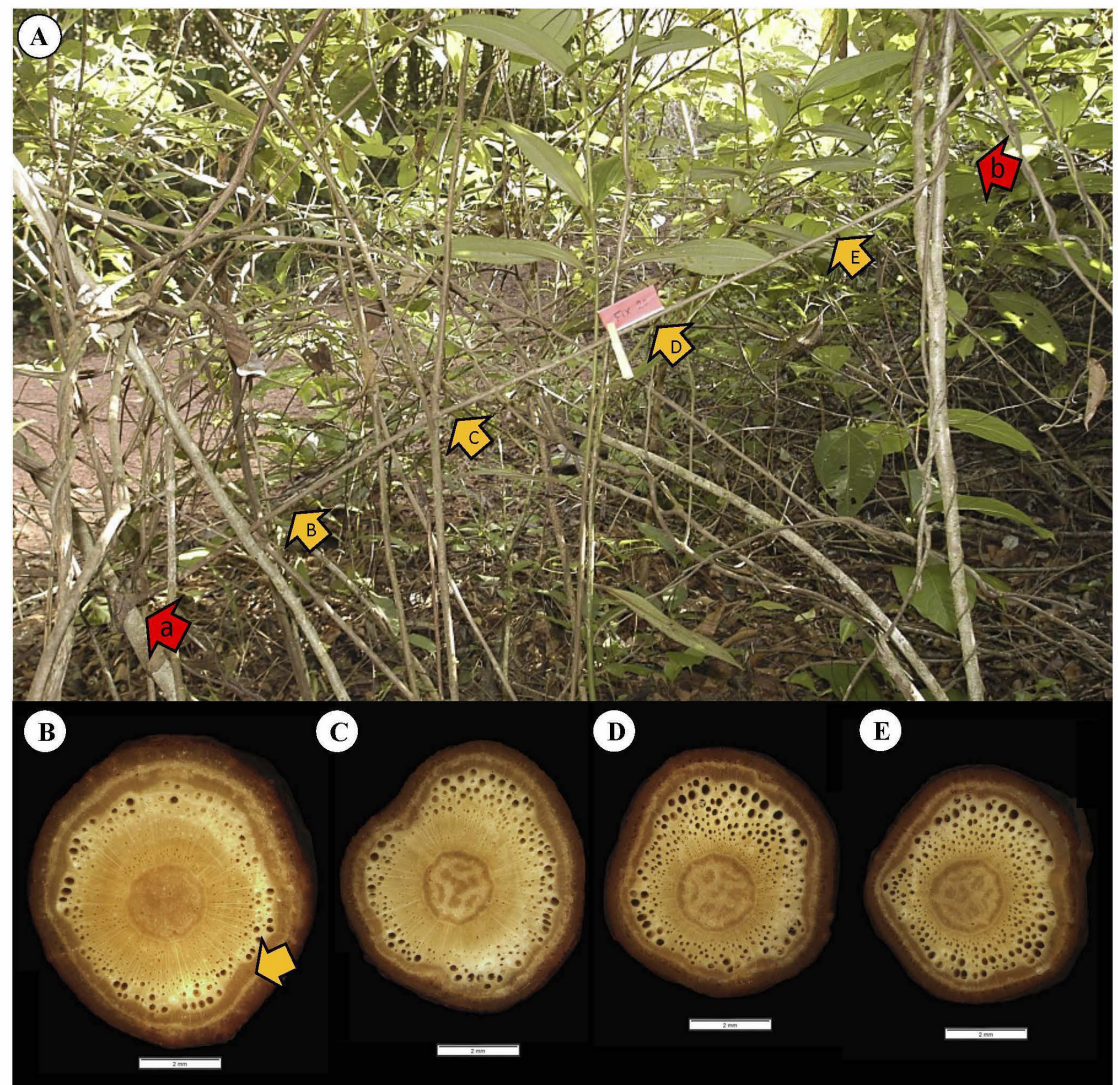
Representative fixed stem of *C guianense*. **(A)** In exact field position at time of collection along with macro-anatomical sections [**(B–E)** and yellow arrows] along the stem. The red arrows a and b indicate the lower and upper points of twining attachment respectively. The diameter of TYPE I wood increases toward the base of the fixed segment indicating the original self-supporting organization. The thickness of TYPE I wood near the upper limit of the segment is very narrow and consistent with (**Figure 12**) that the onset of TYPE II wood has started just following the twining attachment to the upper point of attachment. The development of TYPE II wood appears to be initiated later in the lower part of the fixed stem.

Our pooled data from all kinds of self-supporting to fixed stems indicate that the width of initial TYPE I wood can be very narrow (**Figures 10**–**13**), suggesting that TYPE II wood can be triggered even in very young, narrow searcher stems that connect with a support.

### Fixed stems (construction)

We have emphasized the difficulty of obtaining comparative samples of the stems making up a liana trellis. This is particularly true for the so-called fixed stem category where no stem has undergone the same development under the same environmental conditions. A representative fixed stem of a trellis (fixed by twining below and above) shows a lianoid stem connected at two points to two young saplings about a meter and a half apart (**Figure 13**, red arrows). It is forming a trellis between two robust stems and is continuous and supplying hydraulically the liana trellis above. The pattern of tissue development is consistent with the findings of the main pooled data set as well as the example discussed in the previous climbing and attaching stem (**Figure 12**). The gap between the two supports corresponds to the approximate maximum reach for the species of *c.* 1.5 m. The oldest segment close to the basal attachment point (**Figure 13B**) shows evidence of a broad cylinder of TYPE I wood. This represents the basal part of the original self-supporting axis that has reached from the basal support to the upper support. The cylinder of stiff TYPE I wood tapers toward the top of the fixed segment (**Figures 13B–E**). Interestingly, all segments have developed a layer of TYPE II wood, which is developed significantly occupying a large cross-sectional area especially in the more apical segments. Hence, the pattern of original self-supporting organization and then attachment is consistent with the previous climbing stem example (**Figure 12**) where TYPE II wood was initiated near the apex of the self-supporting stem where there was very little TYPE I wood close to the point of attachment. The example is also interesting because it shows what must have been a self-supporting searcher emerging from the attachment on the lower left (**Figure 13A**, red arrow a). And this is exactly what was seen in (**Figure 13A**) where a narrow searcher continues searching a space AFTER a first attachment. The basal segment and the diameter of the TYPE I wood approximately corresponds to the 5-6 mm rule, prior to the searcher apex reaching the sapling on the right (**Figure 13A**, red arrow b). Following fixation in the trellis, there is no longer any requirement in this stem for TYPE I stiff wood for optimized self-support, and stem development continues indeterminately with TYPE II wood growth.

### Structure-function partitioning

**Figure 14:**
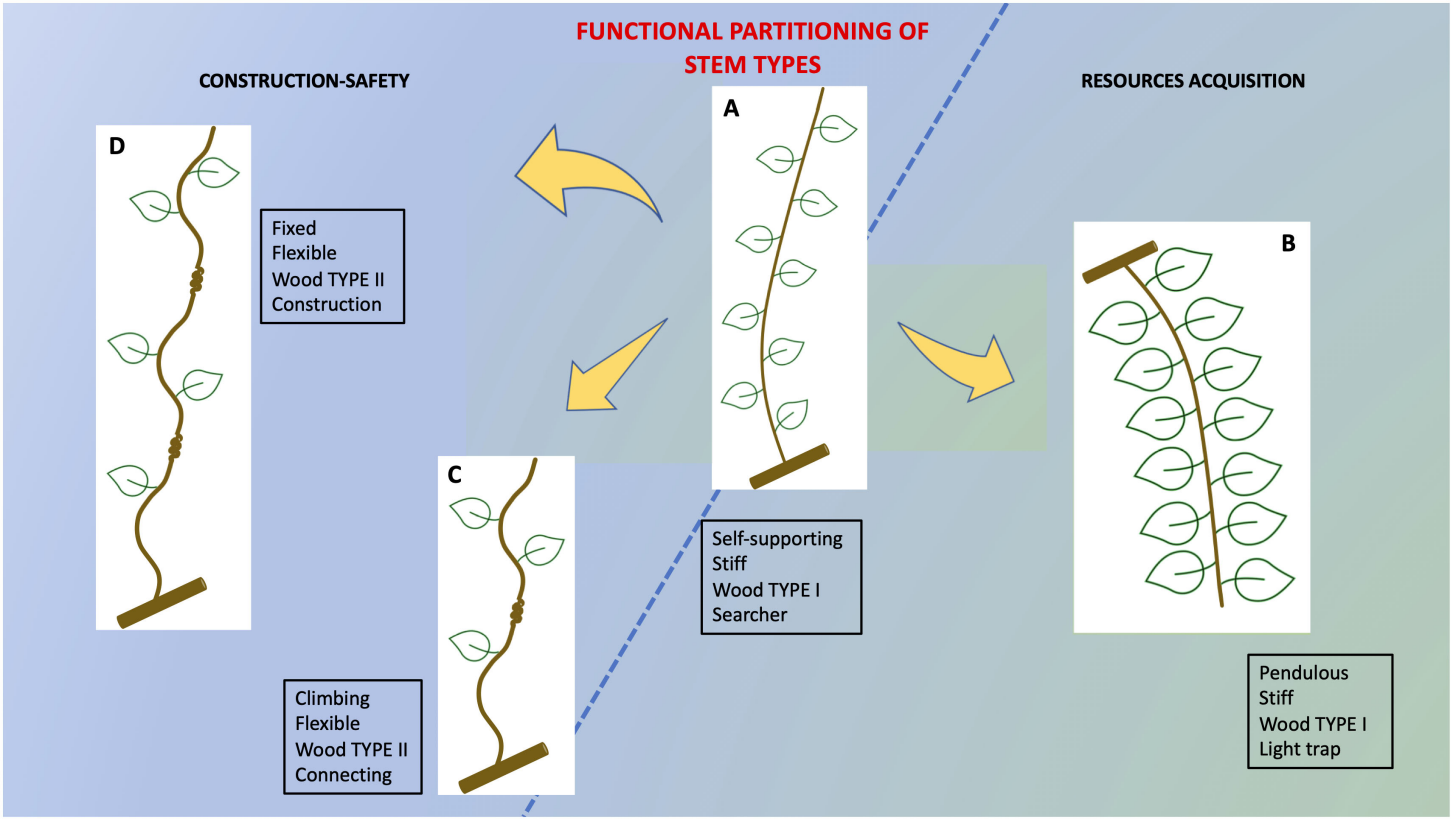
Summary of structural–functional partitioning in trellis stems of *C guianensis*. The overall ontogenetical trend from narrow-stiff to wide stems with reduced stiffness follows recent studies of the plant that have described mechanical changes based on the overall growth and change in size. However, the construction of complex 3D trellis structures that are essential for twining liana canopies to remain aloft and suspended from self-supporting hosts safely requires fine-tuned modifications stops and starts of the stem ontogenetic developmental trajectory. All four stem types are derived from the same cauline organization but developmental “stops and starts” modify specific stem behaviors to **(A)** limit self-supporting growth to a diameter of 5–6 mm. **(B)** Recommissioning self-supporting stems to pendulous leaf-bearing photosynthetic stems of the trellis if a support is not reached **(C)** initiate TYPE II lianoid wood in significant amounts only if attachment to a support is made. **(D)** Enablement of multi attachment (serial fixed points) following the first attachment.

The results suggest that *Condylocarpon guianense* develops a functional partitioning *via* developmental “stops and starts” of the cambial development of the stem to construct a complex 3D trellis (**Figure 14**). We summarize these functional attributes *via* four representative examples (**Figures 10** **–** **14**). Following the young self-supporting stage, there are two possible developmental outcomes: If the young axis reach the threshold of 5–6 mm in diameter and does not find a support, then it buckles, develops more leaves, and becomes adapted to energy production as a pendulous stem. If it successfully attaches to a host support, then it adapts locally near the point of attachment to modify its stem properties *via* TYPE II lianoid wood and the formation of flexible properties that then spread basipetally from the point of attachment. Our observations also showed that attached stems can continue growing as self-supporting searchers following twining and find another support and repeat the process of developing TYPE II wood, resulting in a sequence of fixed plant segments and gradients of TYPE I and TYPE II layered wood. This development of stiff and lianoid wood properties combined with circumnutatory, twining behavior is a relatively simple “strategy” using a simple on-off mechanism to provide mechanical properties that can construct highly complex and seemingly disordered branch systems typical of liana trellis systems.

Of course, trellis structures are subject to many changes because of the environment such as slipping and partial falls. Orientations and physical situations of individual shoots and branches can change. None of the stem segments that we observed showed any consistent “returns” to a self-supporting organization with stiff tissue produced after formation of the entire ring of TYPE II wood. This suggests that, overall, the development described is not reversible. We did notice, especially, among pendulous stems, that partial rings of large diameter vessels were formed and then succeeded by additional dense wood, but we point out that this is possibly more related to the development of leaves in a self-supporting or pendulous stage of growth.

### Toward technical applications

Climbing plants are increasingly being viewed as biological models for new bioinspired technologies in soft robotics (Mazzolai, 2017; Wooten et al., 2018; Wooten and Walker, 2018; Fiorello et al., 2020; Bastola et al., 2021). Some of the potential applications involve additive growth, adaptive sensing, and movement toward supports as well as enabling anchorage and spanning of gaps through unpredictable and heterogeneous environments. Trellis-forming shoots of lianas such as *C. guianense* perform many of the tasks that are consistent with this potential wish list of liana-like behaviors. Recent studies have also explored the advantages of stem braiding—intertwining of climbing plant shoots into multi-stranded functional units (Gallentine et al., 2020). Interaction between individual growing units (stems) and their capability to auto-construct complex structures even within and as part of highly complex 3D environments is a highly desirable task for adaptive growing robotic artefacts. This exploration of trellis-forming in *Condylocarpon* suggests that, in biology, highly complex, mechanically adapted structures at “ecological scales” can develop safe, net-like structures. They achieve this *via* a simple stop-start “decision-making” based both on environmental cues and internal “rules”. As an example of an “internal rule”, the 5- to 6-mm threshold observed in *C. guianense* for self-supporting growth apparently curtails “unwanted” possibly non-adaptive development of big self-supporting stems for the specialist niche that the species has presumably been selected for. Other rules include the stop-start development of flexible wood when the stem encounters a support and its basipetal spread down the stem to transform the previously self-supporting body into something that will likely be highly resistant to damaging failure. All of these developmental and functional processes are intrinsically simple but result in highly complex structures. This simple-to-complex hierarchy of adaptive structural construction is highly desirable for technical self-constructing systems.

## Data availability statement

Data supporting the findings of this study are available at Zenodo, Soffiatti et al. https://doi.org/10.5281/zenodo.7337338.

## Author contributions

NR conceived of the project and carried out field work with Michaël Guéroult. EF and CH developed the anatomical procedures and prepared the material for analysis and a preliminary report of the technical findings. PS and NR analyzed the data and prepared the manuscript. All authors contributed to the article and approved the submitted version.
